# Smoking cessation is related to change in metabolic syndrome onset: A rural cohort study

**DOI:** 10.18332/tid/118232

**Published:** 2020-03-06

**Authors:** Myung-Bae Park, Cheon-Kook Kang, Jung-Kyu Choi

**Affiliations:** 1 Department of Gerontology Health and Welfare, Pai Chai University, Daejeon, Republic of Korea; 2 Department of Health Administration, Baekseok Culture University, Cheonan, Republic of Korea; 3 Institute of Health Insurance and Clinical Research, National Health Insurance Corporation Ilsan Hospital, Goyang, Republic of Korea

**Keywords:** metabolic syndrome, smoking cessation, cohort, rural, health education

## Abstract

**INTRODUCTION:**

Relatively few, mainly cross-sectional, studies have examined the relationship between smoking cessation and metabolic syndrome (MetS). In particular, information on smoking cessation after MetS is limited. This study aimed to investigate the probability of smoking cessation after the onset of MetS.

**METHODS:**

In this study we used cohort data from a rural area of Korea and extracted the data of 1054 smokers who were identifiable at baseline and were followed up. Of these, 1041 individuals were selected. Descriptive statistical analyses were performed to identify the basic characteristics of smokers. Multiple logistic regression was performed to determine the association between changes in MetS and smoking cessation.

**RESULTS:**

The probability of smoking cessation was 1.84 times higher in the newly developed MetS cohort than in the reference group (without MetS at any time point), and it was 1.61 times higher in the persistent MetS cohort than in the reference group, with both probabilities being significant.

**CONCLUSIONS:**

We found that patients with MetS were more likely to quit smoking than those without MetS. However, intervention is still needed, as numerous patients with MetS continued to smoke. Interventions that actively involve medical institutions or organizations are among the most effective approaches to promote smoking cessation in patients with MetS. In particular, women, farmers and current drinkers should be prioritized.

## INTRODUCTION

Tobacco use is a risk for various illnesses and diseases^1,2^. Smoking affects the course of most diseases, including disease related to lungs, kidneys and neck, as well as respiratory and cardiovascular diseases and even influenza^3,4^. The lifespan of smokers is reportedly shortened by an average of 15 years^5^. More than 5 million people die every year from tobacco-related causes. Approximately 500 million current smokers are expected to die due to tobacco-related causes, half of which are essentially middle-aged (35–69 years).

Along with smoking, metabolic syndrome (MetS) is another currently important risk factor for disease. MetS refers to the simultaneous occurrence of abdominal obesity, hypertension, dyslipidemia, and impaired fasting blood glucose in a person, and is known to increase the risk of cardiovascular disease and type 2 diabetes and is related to increased mortality^6,7^. Although the cause of MetS remains unclear, abdominal obesity and insulin resistance are known to be the major pathological causes^8^. Among various environmental factors and lifestyle habits, smoking, lack of exercise and an unbalanced diet reportedly have a significant impact^9,10^.

Smoking and MetS are known to be closely related^11^. Smoking is considered to increase and smoking cessation to decrease the incidence of MetS^12-14^. However, most studies on the relationship between smoking and MetS have primarily compared smokers with non-smokers or have focused on the relationship between smoking and MetS^15^. There have been relatively few, mainly cross-sectional, studies on the relationship between smoking cessation and MetS. Smoking cessation has a positive effect on almost all aspects of health, including MetS. It reduces blood clotting, and the heart can supply blood and oxygen to the whole body with less burden, and reduces the risk of myocardial infarction in individuals with other risk factors, especially hypertension, elevated blood cholesterol, being overweight, and diabetes^16^. Therefore, for patients with MetS who are at high risk of serious diseases such as stroke and ischemic heart disease, smoking cessation is necessary for survival^17^, and smokers with MetS are strongly recommended to stop smoking^18,19^.

However, many patients with MetS will continue smoking, and rural residents generally have worse health-related behavior, such as smoking^20^. The prevalence of MetS and chronic diseases is increasing, and rural areas are more severe^20-22^; thus, added attention and consideration is needed for smokers with MetS. Nevertheless, information on MetS after smoking cessation is limited. Previous studies have shown that the occurrence of cancer enhances smoking cessation, but many patients, nevertheless, still smoke^23^. Moreover, it is expected that MetS patients are still in a similar situation. The aim of this study was to investigate the probability of quitting smoking after the onset of MetS in a rural cohort in Korea.

## METHODS

### Data source

We used cohort data from the Korea Genome and Epidemiology Study (KoGES) provided by the Korea Centers for Disease Control & Prevention (KCDC). KoGES is divided into community-based, urban-based, rural-based, twin, and family cohorts. The cohort group was designed for individuals aged 40–65 years. This study used rural cohort data from two regions of South Korea. Baseline surveys were conducted between 2005 and 2011, and the first follow-up was conducted between 2007 and 2014. As of March 2019, only data from the first follow-up are available. Because the data are publicly available, ethical approval was not required for this study. All procedures followed were in accordance with the ethical standards of the responsible committee on human experimentation (Institutional Review Board of National Health Insurance Medical Center and Pai Chai University). This study was carried out in accordance with the ethical standards of the Helsinki Declaration (1975). The data reported in this article (Korea Genome and Epidemiology Study) are publicly available for download and analysis:

(http://www.cdc.go.kr/NIH_NEW/contents/ NihKrContentView.jsp?cid=26458&viewType=C DC&menuIds=HOME005-MNU2016-MNU1010MNU1543).

The cohort survey area comprised several counties; the baseline survey was surveyed per county for about 6 months, and the follow-up survey was conducted in the same order. This cohort study included health surveys and physical measurements. Health surveys included questions on smoking, drinking, and physical activity. Physical measurements included height and body weight, diabetes screening, vital signs, body composition, bone mineral density, radiography, ultrasonography. Blood and urine tests were performed and the samples were stored in the cohort epidemiological information system after analysis. The samples were stored in the deep freezer of the Korean biobank network for genetic analysis. All surveys, including the health survey physical measurements were performed by nurses regularly trained for the purpose of the surveys.

### Subject selection

For this study, the data of 1054 smokers who could be identified at baseline were extracted, and this size of sample had a 95% confidence interval (CI) within 3 standard deviations (SDs) from the mean.

**Figure 1:**
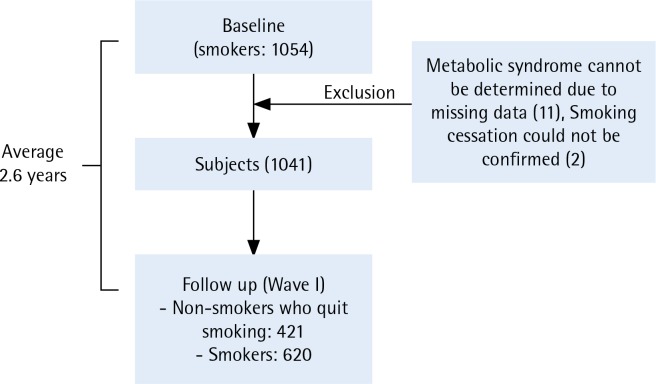
Flow diagram for subject follow-up

Of these smokers, 1041 were selected as final study participants, excluding 11 for whom MetS could not be determined due to missing data and 2 for whom smoking cessation could not be confirmed because they did not provide a response regarding smoking in the follow-up survey. Among these 1041 individuals, 421 successfully stopped smoking. The average duration from baseline to follow-up was 2.6 years (Figure 1). Individuals were divided into the following four groups: 1) individuals who did not have MetS both at baseline and at the follow-up (non-MetS), 2) individuals who had newly developed MetS by the follow-up (newly developed-MetS), 3) individuals who had MetS both at baseline and at the follow-up (continued-MetS), and 4) individuals who had MetS at baseline but had resolved by the followup time (Former-MetS).

### Variables and outcomes

Smoking was confirmed by a self-reported survey. This study defined smokers as those ‘who smoked more than 100 cigarettes during their lifetime’. Moreover, among the respondents who stated that they were currently smoking at the baseline, those who reported that they no longer smoked at the follow-up were defined as participants who had stopped smoking. In order to prevent a response error, we asked an additional question on whether cigarette smoking had currently been stopped. MetS was defined according to the Asian standard of the International Diabetes Federation, such that if three or more of the following five items were present, the individual was considered to have MetS^24^: 1) waist circumference ≥90 cm for men and ≥80 cm for women; 2) serum triglyceride level ≥150 mg/dL or diagnosis of dyslipidemia; 3) serum high-density lipoprotein cholesterol level <40 mg/dL for men and <50 mg/dL for women; 4) systolic blood pressure >130 mmHg or diastolic blood pressure >85 mmHg or diagnosis of hypertension; and 5) fasting plasma glucose ≥100 mg/dL or diagnosis of diabetes mellitus. In addition, the results were adjusted for basic socioeconomic factors including sex and age, and also alcohol consumption as it is closely related to MetS and smoking^13^,^25^ and important in determining the correlation between current drinking status and smoking status. Those who answered that they currently drank alcohol at follow-up were defined as current-drinkers, while ex-drinkers included both those who used to be drinkers but stopped or those who were not drinkers at baseline and in wave 1. Finally, the duration (months) between the two points was used as a control variable since the participants differed between the baseline and follow-up.

### Data analysis

First, descriptive statistics were performed to identify the basic characteristics of smokers. From the baseline to the follow-up period, and changes in smoking status were observed according to changes in MetS. In addition, multiple logistic regression analysis was performed to determine whether the participants had quit smoking according to changes in MetS. For analysis, we used SAS for Windows 9.4.

## RESULTS

### Participants’ characteristics

The sociodemographic characteristics of the 1041 participants were as follows. At the follow-up point, the average age of the participants was 55.7 years (SD = 8.3). There were 997 men (95.8%) and 44 women (4.2%). Regarding the employment status, 539 (51.8%) were working in the agricultural sector, and 502 (48.2%) had non-agricultural occupations. Of all participants, 741 (71.1%) were current drinkers, and 435 (41.7%) had MetS. For reference, 751 (72.0%) were drinkers and 481 (46.1%) had MetS at baseline (Table 1).

**Table 1:** General characteristics of the study population, Korea (N=1041 )

*Variables*	Categories*	*n*	*%*
**Age** (years) mean (SD)		1041	55.7 (8.3)
**Sex**	Male	997	95.8
	Female	44	4.2
**Job status**	Agriculture	539	51.8
	Others	502	48.2
**Alcohol drinking**	Current non-drinker (Ref.)	740	71.1
	Ex-drinker	49	4.7
	Current drinker	252	24.2
**Prevalence of metabolic syndrome**		435	41.8

* Based on wave 1.

### Changes in smoking status following the onset of MetS

Of the 1041 smokers at baseline, 421 (40.5%) were registered as non-smokers (stopped smoking) at follow-up. Both at baseline and follow-up, 162 (36.6%) participants who did not have MetS had stopped smoking. At follow-up, 118 patients were newly classified with MetS; of these, 47 (39.8%) had stopped smoking. Among the 317 participants with MetS at both baseline and follow-up, 150 (47.3%) had quit smoking. There were 163 participants at baseline who had MetS but were MetS-free at follow-up, of which 62 (38.0%) had stopped smoking (Table 2).

**Table 2:** Smoking status according to the presence of metabolic syndrome in a rural cohort, Korea (N=1041 )

	Nonsmoker* (quit smoking) n	*%*	Current smoker* n	*%*
**Participants**	421	40.5	620	59.5
**Metabolic syndrome status**				
Non-MetS (n=443)	162	36.6	281	63.4
Newly developed-	47	39.8	71	60.2
MetS (n=118)				
Continued-MetS (n=317)	150	47.3	167	52.7
Former-MetS (n=163)	62	38.0	101	62.0

Non-MetS: individuals who did not have MetS, both at the beginning and the followup of the study. Newly developed-MetS: individuals who newly developed MetS at the time of follow-up. Continue-MetS: individuals who had MetS, both at the beginning and the follow-up of the study. Former-MetS: individuals who had MetS at baseline but had resolved by the follow-up point.

* Based on follow-up point.

### Analysis of smoking-cessation patterns by multiple logistic analysis

Model 1 shows the relationship between smoking cessation and variables that may affect MetS incidence, such as age, sex, job status and current drinking status, that were adjusted for (Table 3). The probability of smoking cessation was 1.36 times higher among those with non-agricultural occupations than among those with agricultural occupations, and 0.629 times lower among those who were current drinkers than among non-drinkers. In Model 2, only change in MetS was included, while the group without MetS at both baseline and follow-up was used as the reference. Compared to the reference group, those who had newly-developed MetS had a 1.79 times higher cessation rate, while the group that continued to have MetS also had a 1.65 times higher cessation rate; these were statistically significant. Model 3 included all variables (major variables adjusted for and MetS changes). The probability of smoking cessation was 0.49 times lower in women than in men, 1.34 times higher in participants with non-agricultural occupations than in those with agricultural occupations, and 0.64 times lower in current drinkers than in non-drinkers. Compared with the reference group (without MetS at any point), the cohort with newly-developed MetS had a 1.84 times higher probability of smoking cessation, and the cohort that continued to have MetS had a 1.61 times higher probability; these were statistically significant. Conversely, within the cohort that had MetS at baseline but not at follow-up, there was no statistically significant difference compared with the reference group (Table 3).

**Table 3:** Relationship between metabolic syndrome and smoking cessation assessed using multiple logistic regression within a rural cohort, Korea (N=1041 )

		*Model 1*		*Model 2*		*Model 3*	
*Variables*	*Categories*	*Exp (b)*	*95% CI*	*Exp (b)*	*95% CI*	*Exp (b)*	*95% CI*
**Age**		1.012	0.995–1.028			1.011	0.995–1.028
**Sex**	Male (Ref.)	1					1
	Female	0.527	0.268–1.038			**0.492**	**0.248–0.977**
**Job status**	Agriculture (Ref.)	1					1
	Others	**1.360**	**1.040–1.776**			**1.337**	**1.021–1.750**
**Alcohol drinking**	Current non-drinker (Ref.)	1					1
	Ex-drinker	0.811	0.430–1.531			0.832	0.440–1.574
	Current drinker	**0.629**	**0.461–0.856**			**0.640**	**0.469–0.873**
**Metabolic syndrome status**	Non-MetS (Ref.)			1			1
	Newly developed-MetS			**1.787**	**1.081–2.952**	**1.838**	**1.107–3.052**
	Continued-MetS			**1.649**	**1.099–2.473**	**1.612**	**1.065–2.439**
	Former-MetS			1.012	0.648–1.578	1.038	0.661–1.632

Non-MetS: individuals who did not have MetS, both at the beginning and the follow-up of the study. Newly developed-MetS: individuals who newly developed MetS at the time of follow-up. Continue-MetS: individuals who had MetS, both at the beginning and the follow-up of the study. Former-MetS: individuals who had MetS at baseline but had resolved by the follow-up point. * The duration between the baseline and follow-up was adjusted.

## DISCUSSION

This prospective study was conducted to investigate the association between smoking cessation and the onset of MetS. Patients with MetS were more likely to stop smoking. The findings revealed associations between MetS and smoking cessation. The factors associated with smoking cessation in our study were sex, employment status, drinking status, and onset of MetS. The probability of smoking cessation was significantly lower in women and in current drinkers. Moreover, the probability of smoking cessation was significantly higher in participants with non-agricultural occupations.

The guidelines of the American Diabetes Association and American Heart Association classify patients with diabetes who smoke and have hypertension, which are major causes of MetS, as those with the highest risk of developing heart disease^26^. It is known that when patients with chronic conditions smoke, it increases the incidence of cardiovascular disease (CVD), but that the incidence decreases by 10–50% if they stop smoking^27^. An interesting question is whether the onset of disease will motivate smokers to stop smoking. Previous studies have reported that some patients were unable to stop smoking even after a stroke, and 30% of the patients did not attempt to stop smoking within three years^28^. In the Twardella et al.^29^ study, the diagnosis of stroke and diabetes was found to affect smoking cessation, but the probability of smoking cessation decreased over time after the diagnosis. Although there was no statistically significant difference in this study, the cessation rate in the continued-MetS group was slightly lower than that in the newly developed-MetS group. In the present study, after approximately 2.6 years of follow up, the group that had newly developed MetS by the time of the follow-up and the continued-MetS group had a 61–84% higher probability of smoking cessation compared to the non-MetS group. No separate interventions (such as smoking cessation education for improved health outcome) were provided (even if there were other diseases as well as MetS) for the participants in this study, as a cohort. However, the physical examinations, including the blood and urine tests results, were sent to the participants by mail, while those with MetS were provided with simple recommendations for smoking cessation such as reducing the intake of alcohol and increasing physical activity. Therefore, it is possible that the participants were aware of their MetS status and decided to implement healthy lifestyle changes. In addition, many patients with MetS were also diagnosed with a chronic disease such as diabetes, hypertension, or dyslipidemia. They were also more likely to be urged to quit smoking while consulting with their doctors from time to time. However, this only signifies that the groups had a higher probability of smoking cessation compared to the healthy reference group, and even in this study, more than half of the patients with MetS continued smoking. As the present study is based on a cohort drawn from rural areas, we cannot generalize the results; nevertheless, considering previous studies on smoking cessation following the onset of disease^28,29^, we could expect with high probability that many individuals would continue smoking even after the onset of MetS. This indicates that the highest risk group (patients with MetS who smoke) for severe diseases such as CVD is quite numerous.

In view of the above, smoking cessation interventions are required for such individuals. Desire and intention to stop smoking are the most important motivators for smoking cessation^30^. However, as it is considerably challenging to stop smoking unaided^31^, intervention for smoking cessation is necessary. The occurrence of MetS is a negative event, but, paradoxically, offers a window of opportunity for smoking cessation. In particular, the average age of the subjects in this study was 56 years, which is relatively high. As older individuals generally have erroneous information regarding smoking and more fixed attitudes toward smoking^32^, older people are less likely to stop smoking^33^. Therefore, from a life-cycle perspective, interventional programs should be designed to provide middle-aged or older people with awareness and motivation for smoking cessation^34^. However, rural health care services are generally more vulnerable than those in urban areas^35-38^. To overcome this health inequality, it is necessary to urge the local community to make the most of existing resources. In rural areas, there is less opportunity to participate in health education programs than in urban areas^35,37,39^. Therefore, there is a need to improve the benefits of health education through more effective interventions in rural areas.

In addition, the elderly or patients with MetS frequently use medical-care services^40^. Accordingly, the healthcare management of patients with MetS through the community public health center has been shown to be effective at promoting positive lifestyle changes, including smoking cessation^41^. Moreover, Rigotti et al.^42^ concluded that a smoking cessation intervention for hospitalized smokers was effective. In terms of setting an approach, interventions that actively involve medical institutions or organizations are among the most effective means of promoting smoking cessation in patients with MetS. According to previous studies on interventions using the Ajzen^43^ ‘Theory of Planned Behavior’, providing guidance on attitude, subjective norms, perceived behavioral control, and intention, has been verified to be sufficiently effective to induce smoking cessation^44,45^. For smoking cessation, it might be helpful to prescribe medication and intensive counselling, but intensive counselling is not always more effective^46^. Since increasing awareness on smoking cessation through motivational programs is effective^34^, if a systematic intervention is provided via a simple program to offer motivation by medical doctors, nurses, or health educators when patients with MetS visit public health centers or medical clinics, it could serve as a powerful health protection program for patients with MetS who smoke. Although this study was not designed to determine whether smoking cessation reduces MetS, previous cohort studies have shown that smoking cessation leads to a higher probability of MetS resolution^47^. Therefore, smoking cessation interventions for patients with MetS are absolutely necessary for the prevention and treatment of MetS, *per se*, as well as the prevention of serious diseases such as CVDs. Finally, due to geographical and socioeconomic conditions, these services are not available in all regions. Therefore, it is necessary to actively utilize alternative approaches as a means for health education. Intervention through the Internet, mail, and telephone, has been effective^48-50^.

## Limitations

There are some limitations to our study. First, the study included individuals who live in rural communities, and thus the sample is not representative of the national population. The cohort was predominantly male, and by extension, not representative of those with MetS in other populations. Therefore, the results may not be representative of the MetS population as a whole. Second, no information was available on the long-term effect of smoking on health. This information is necessary in order to provide smoking cessation information to patients with MetS. In addition, false responses regarding smoking and drinking may have been given during face-to-face interviews for social desirability reasons^51^. Finally, there may have been changes in MetS between baseline and follow-up, but it was not possible to discern this based on the available data. Despite these limitations, a key advantage of the present study was the examination of a prospective association between MetS and smoking cessation and such evidence-based results can be used to design new policies related to smoking cessation adoption and development. Further research should examine how the incidence and prevalence of MetS may affect smoking cessation in the long-term.

## CONCLUSIONS

Smoking cessation has been strongly recommended for MetS patients. However, few studies have examined whether patients with MetS stop smoking. Our study found that smokers with MetS overall continue to smoke. In addition, it was confirmed that the occurrence of MetS may be a motivator for smoking cessation. In particular, among the patients with MetS, women, farmers and current drinkers had the lowest smoking-cessation rates and should be prioritized for smoking cessation interventions.
